# Positive Association of Vitamin D Receptor Gene Variations with Multiple Sclerosis in South East Iranian Population

**DOI:** 10.1155/2015/427519

**Published:** 2015-01-20

**Authors:** Mehrnaz Narooie-Nejad, Maryam Moossavi, Adam Torkamanzehi, Ali Moghtaderi

**Affiliations:** ^1^Genetics Department, Zahedan University of Medical Sciences, Zahedan 9816743463, Iran; ^2^Genetic of Non-Communicable Disease Research Center, Zahedan University of Medical Sciences, Zahedan 9816743463, Iran; ^3^Department of Biology, Sistan and Baluchestan University, Zahedan 98155-987, Iran; ^4^Department of Neurology, Zahedan University of Medical Sciences, Zahedan 9816743463, Iran

## Abstract

Among the factors postulated to play a role in MS susceptibility, the role of vitamin D is outstanding. Since the function of vitamin D receptor (VDR) represents the effect of vitamin D on the body and genetic variations in VDR gene may affect its function, we aim to highlight the association of two VDR gene polymorphisms with MS susceptibility. In current study, we recruited 113 MS patients and 122 healthy controls. TaqI (rs731236) and ApaI (rs7975232) genetic variations in these two groups were evaluated using the polymerase chain reaction-restriction fragment length polymorphism (PCR-RFLP) technique. All genotype and allele frequencies in both variations showed association with the disease status. However, to find the definite connection between genetic variations in VDR gene and MS disease in a population of South East of Iran, more researches on gene structure and its function with regard to patients' conditions are required.

## 1. Introduction

Multiple sclerosis (MS) (OMIM126200) is a chronic inflammatory, neurodegenerative, demyelinating, and accumulating debilitative disease of the central nervous system (CNS) with a complex etiology that affects over 2.1 million people worldwide [1]. Like most complicated disorders, genetic and environmental elements play a chief role, whether in synergistic or in independent manners [2]. Thus far, many genes have been identified in predisposing to MS disease. Genome-wide association studies (GWAS) suggested more than 20 loci in MS susceptibility, including vitamin D receptor (VDR) gene [3, 4]. According to the recent studies of the role of vitamin D in MS, this association seems reasonable. Vitamin D as a secosteroid hormone is predominantly produced from 7-dehydrocholesterol in the skin during the exposure to ultraviolet (UV) radiation of the sunlight and by dietary intake as well. The active form of vitamin D (1,25-dihydroxyvitamin D_3_) is identified as a ligand for VDR.

The VDR is an intracellular polypeptide that belongs to the steroid-thyroid-retinoid acid receptor superfamily. It binds to the DNA in target cells as VDR/VDR homodimers or VDR/RXR heterodimers in order to initiate the synthesis of special RNA encoding proteins that activates a range of biological functions or mediates suppression of gene transcriptions [5–7].

VDR gene is mapped on chromosome 12q12-14 and contains 9 exons and 8 introns [8]. Some studies suggested that the role of vitamin D in the pathogenesis of MS is through its strong immune-modulating effect [9, 10]. Furthermore, increasing data have provided facts that 1,25-(OH)_2_D_3_ is involved in brain function. Hence, the nuclear receptor for 1,25-(OH)_2_D_3_ has been concentrated in neurons and glial cells in order to operate its function [11]. Vitamin D exerts a direct impact on T-lymphocyte proliferation notably by upregulating T regulatory cells in the presence of active form of vitamin D [12], along with reduction of proinflammatory cytokine, interferon-gamma, production [13]. It has been found that high amounts of IFN-*γ* exist in sera, plasma, and lesions of individuals affected with MS [14]. Furthermore, vitamin D_3_ inhibits B cell proliferation by increasing the cell cycle check point, p27, and induces apoptosis in activated B cells, thus reducing antibody production via its nuclear hormone receptor [15]. Neuroprotective and immunomodulatory influences of this hormone have been explained in several experimental models as well. Alternative studies reported that MS individuals have also low serum levels of vitamin D and this condition is manifested by increasing rates of bone fractures happening in MS patients in comparison to healthy people [16]. Investigations on an MS animal model, experimental autoimmune encephalomyelitis (EAE), have shown an absolute inhibition of disease course following the injection of the biologically active form of vitamin D [17]. Moreover, studies of VDR knockout mice show that absence of VDR is crucial for EAE activity [18]. By these findings, VDR and its ligand show relative immunosuppressive and anti-inflammatory role. Several studies have confirmed the variation of genes in different individuals [19]. The VDR polymorphisms have been reported to be linked with an increased risk of several autoimmune diseases including MS [20–22]. Specific variations of the VDR gene may alter vitamin D function and metabolism and have been investigated in studies evaluating the function of vitamin D on MS [23]. These findings suggest that allelic variation of the VDR gene may at least partially represent the genetic component of MS disease. By these genetic findings and the probable importance of the VDR in MS, we sought to investigate the relationship between the VDR gene two single nucleotide polymorphisms, TaqI (rs731236) and ApaI (rs7975232), and MS susceptibility in South East Iranian population.

## 2. Material and Methods

### 2.1. Study Population

A total of 235 subjects were recruited in this study, including 113 MS patients clinically diagnosed with MS according to McDonald's criteria [24] and 122 aged-matched controls free from any neurologic and systemic disorder. All patients referred to Imam Ali University Hospital in Zahedan, Sistan and Baluchistan province of Iran. All participants provided informed consent according to the Declaration of Helsinki and accepted codes of the university ethics committee.

### 2.2. Genotyping of VDRG Polymorphisms

Genomic DNA was extracted from the whole blood samples provided from each subject by using salting out protocol [25]. The ApaI and TaqI polymorphisms were identified using the polymerase chain reaction-restriction fragment length polymorphism (PCR-RFLP) technique. The primers sequences which were used to amplify the target fragments containing the polymorphisms to be studied are shown in Table 1. The annealing temperature for ApaI and TaqI single nucleotide polymorphisms (SNP) was 68°C and 65°C, respectively. The ApaI and TaqI enzymes were used to distinguish the related SNPs. Digested products were separated by electrophoresis on a 2.5% agarose gel and visualized by ethidium bromide staining.

### 2.3. Statistical Analysis

The distribution of genotypes and alleles in patients and healthy controls was evaluated for deviation from Hardy-Weinberg Equilibrium. Data were analyzed by using the statistical software SPSS 18 (SPSS, Chicago, IL). Differences in genotypic and allelic distribution between patients and controls were determined by independent sample *t*-test and Chi-square (*χ* ^2^) test. The odds ratio (OR) and 95% confidence intervals (CI) were also estimated. Values of *P* < 0.05 were considered statistically significant.

## 3. Result

113 MS patients (88 women and 25 men) with a mean age of 32.4 ± 8.9 and 122 unrelated healthy controls (94 women and 28 men) with a mean age of 30.8 ± 10.2 were recruited to our study. Age and sex adjustment were performed between the two groups. The mean age of onset for these patients is 28.6 ± 8.6. Genotype frequencies for TaqI and ApaI polymorphisms hold the Hardy-Weinberg equilibrium (*P* > 0.05).

As it has been shown in Table 2, positive associations with MS were found in CC (*P* values < 0.0001; OR = 313, 95% CI in 65.5–1500) and TC genotypes (*P* values < 0.0001; OR = 17.7, 95% CI in 7.6–40.9) and negative association was found with the disease in the TT genotype for TaqI (rs731236) polymorphism. The frequency of C allele of TaqI polymorphism was significantly higher in patients than in controls (*P* values < 0.0001; OR = 18.9, 95% CI in 11.6–30.3); it can be concluded that allele C showed positive association and allele T showed negative association with MS. In the ApaI single nucleotide polymorphism investigation (rs7975232), homozygote genotype CC was significantly higher in patients (*P* = 0.036; OR = 3.4, 95% CI in 1.1–10.4) in comparison to controls. However, the AA genotype frequency indicated negative associations with MS too. Furthermore, allele frequency of C was in positive association with MS (*P* values = 0.019). The calculated value of *P* for AC genotype in ApaI was around the significant area (*P* = 0.056). For better evaluation, more samples are needed to clarify this relationship, which was not available to us.

## 4. Discussion 

Several studies have been conducted on the impact of environmental elements on the rate of MS morbidity [26]. It appears that the frequency of vitamin D deficiency has a significant role in causing the disease [27]. So far, several publications are released regarding association of MS with vitamin D concentration in serum and cerebrospinal fluid. But with the conflicting results obtained, further investigation is needed [27–30]. Since the role of vitamin D in MS is still the subject of investigations, it is reasonable to evaluate the role of the VDR in MS susceptibility. In fact, the function of VDR represents the effect of vitamin D in the body and genetic variations in VDR gene may affect its function. In this regard, 113 MS patients and 122 matched controls in Sistan and Baluchestan province were genotyped for two SNPs, ApaI and TaqI, in VDR gene. This is approximately a distinct population in South East of Iran, with an intermediate prevalence of MS, along with a fast growing incidence rate [31, 32]. Although these variations do not cause a change in protein structure, their proximity to the 3′UTR may affect the translation process or signaling pathway. One study concluded that the gene expression in C (mutant) allele is 30% less than that in T (wild) allele in TaqI variation [33]. In current study, all genotype and allele frequencies in both variations showed association with the disease status. These results are in contrast with the results obtained from Greece [23], USA [19, 33], and Netherlands [34], which believe that there is no association between VDRG polymorphisms and MS disease. Compared to these studies, other surveys in Japan [22, 35], Australia [36], and UK [37] have shown that there are associations between VDRG polymorphisms and MS. It is clear that, with the present data, a definite conclusion cannot be reached in this area.

Note that, in this province, there are no significant differences in the concentration of 25-OH-D3 calcium levels in the serum and cerebrospinal fluid (CSF) in MS patients compared with the control group [28].

Although we could not yet definitely emphasize the role of polymorphisms on VDR function, consider that the variation in vitamin D receptor may affect the ligand-receptor affinity or signaling pathway [38] or gene expression [33] and so indirectly affect the function of vitamin D.

If these polymorphisms are nonfunctional, the possibility arises that these polymorphisms are in linkage disequilibrium (LD) with other factors that affect gene function [38].

However, to find the actual relationship between genetic variations and MS disease, extensive research on gene structure and its function in regard to patients' conditions is required.

## Figures and Tables

**Table 1:** Primer sequences for ApaI and TaqI polymorphisms.

ApaI (rs7975232)	F: 5′-**AGAGCATGGACAGGGAGCAAGGCCAGGCAG**-3′
	R: 5′-**GCGCAGGTCGGCTAGCTTCTGGATCATC**-3′

TaqI (rs731236)	F: 5′-**GGGACGATGAGGGATGGACAGAGC**-3′
	R: 5′-**GGAAAGGGGTTAGGTTGGACAGGA**-3′

**Table 2:** Genotype and allele frequencies of TaqI (rs731236) and ApaI (rs7975232).

SNP	Genotype/allele	Patients	Controls	Odds ratio	*P* value	Power study
		*n* = 113	*n* = 122	(95% CI)		(%)
TaqI (rs731236)	TT, *n* (%)	9 (8)	94 (77)	1		100
	TC, *n* (%)	44 (39)	26 (21.4)	17.7 (7.6–40.9)	**<0.0001**	80.54
	CC, *n* (%)	60 (53)	2 (1.6)	313 (65.5–1500)	**<0.0001**	100
	T, *n* (%)	62 (27.4)	214 (87.7)	1		
	C, *n* (%)	164 (72.6)	30 (12.3)	18.9 (11.6–30.3)	**<0.0001**	100

ApaI (rs7975232)	AA	40 (35.4)	61 (50)	1		56.61
	AC	62 (54.9)	56 (45.9)	1.7 (1–2.9)	0.056	23.34
	CC	11 (9.7)	5 (4.1)	3.4 (1.1–10.4)	**0.036**	29.81
	A	142 (62.8)	178 (73)	1		
	C	84 (37.2)	66 (27)	1.6 (1.1–2.4)	**0.019**	32.77

TaqI genotypes (TT = wild type; TC = heterozygote; CC = mutant type); ApaI genotypes (AA = wild type; AC = heterozygote; CC = mutant type).
